# Effects of dexmedetomidine on dynamic lung compliance in general anesthesia with desflurane: A randomized controlled study

**DOI:** 10.1016/j.heliyon.2023.e16672

**Published:** 2023-05-24

**Authors:** Xiaoli Wang, Chao Gong, Yi Zhang, Shitong Li, Lina Huang, Lianhua Chen

**Affiliations:** a Department of Anesthesiology, Shanghai General Hospital of Nanjing Medical University, No. 650, New Songjiang Road, Shanghai, 201620, China; b Department of Anesthesiology, Shanghai General Hospital, Shanghai Jiao Tong University, No. 650, New Songjiang Road, Shanghai, 201620, China

**Keywords:** Desflurane, Dexmedetomidine, Dynamic lung compliance, Hemodynamics, Oxygenation index

## Abstract

**Objective:**

The aim of this study was to evaluate the effect of dexmedetomidine on lung compliance in patients under general anesthesia with desflurane.

**Methods:**

This prospective, randomized, double-blind, controlled trial included 51 patients who received general anesthesia undergoing lower limb fracture surgery. Participants were assigned to either the experimental (loading dose of 0.25 μg/kg dexmedetomidine over 10 min, followed by a maintenance dose of 0.3 μg/kg/h until the end of the surgery) or control (0.9% saline) group. Anesthesia was maintained with desflurane, analgesics and muscle relaxants. The two groups were compared for hemodynamic parameters, dynamic lung compliance, oxygenation index, and postoperative complications.

**Results:**

While dynamic lung compliance showed no significant difference between the two groups at T1 (*P* = 0.321), it was significantly higher in the experimental group at all other time points (all *P* < 0.001). In the control group, Cdyn at T4, T5, T6, and T7 were lower than that at T1 (*P* = 0.032, 0.043, 0.032 and 0.018, respectively). There were no significant between-group differences in the mean arterial pressure and heart rate. Compared to the control group, the experimental group had a higher oxygenation index at T1 (*P* < 0.001), T2 (*P* < 0.001), T3 (*P* < 0.001), T4 (*P* = 0.02), and T5 (*P* = 0.016) and significantly lower peak airway pressure at all time points (all *P* < 0.001). Both groups had comparable postoperative outcomes.

**Conclusions:**

Dexmedetomidine infusion at a loading dose of 0.25 μg/kg and maintenance dose of 0.3 μg/kg/h improved dynamic lung compliance in patients receiving desflurane during general anesthesia.

## Highlights

• Lung compliance in patients under general anesthesia should be improved.
• Dexmedetomidine improved lung compliance and oxygenation in patients on desflurane.

## 1 Introduction

The volatile anesthetic desflurane has the lowest blood gas distribution coefficient, resulting in a rapid offset of its effect [1,2]. However, the airway response to desflurane may lead to cough, breathlessness, laryngospasm, high blood pressure, and increase secretions and heart rate (HR). Satoh et al. [3] revealed that a 2.0 minimum alveolar concentration (MAC) of desflurane increased airway resistance and decreased lung compliance, whereas sevoflurane had lower effects on both. Nyktari et al. [4] found that maintaining 1.0 MAC desflurane did not affect airway resistance compared to sevoflurane and isoflurane, whereas 1.5 MAC desflurane increased airway resistance. Therefore, increasing the concentrations of desflurane is not advisable for alleviating bronchospasm. Currently, the use of desflurane is limited in patients with a high airway response because of adverse airway reactions. The obvious changes in the respiratory system mainly manifest as increasing residual volume, unchanged total lung capacity, and lower lung compliance, coupled with the effects of general anesthesia caused by blocked gas exchange. Improving lung compliance and avoiding high airway resistance during general anesthesia with desflurane has become a priority in clinical applications.

Dexmedetomidine (Dex) is a highly selective α_2_-adrenergic receptor agonist widely used in the intensive care unit (ICU) to reduce anxiety and for surgical sedation. It acts as a sedative-hypnotic, mainly through activation of α_2_-adrenergic receptors of the presynaptic and postsynaptic central venous system [5]. In a study with 37 healthy volunteers, Dex slightly increased the partial pressure of carbon dioxide, reduced minute ventilation volume, and minimally altered the ventilation frequency after a 2-min injection of 2 μg/kg dexmedetomidine [6]. Bronchodilators mainly act on β-adrenoceptors in the bronchial smooth muscles. Although α_1_- and α_2_- adrenoceptors are found in the bronchial mucosa and ganglia, their bronchial effects have not yet been confirmed. However, some studies have revealed that Dex can reduce pulmonary shunts in healthy patients [7]. Moreover, some animal studies have shown that Dex can effectively inhibit bronchoconstriction induced by histamine release [8].

Because of the different methods and concentrations used in previous studies, there were varying views on the effects of desflurane on lung mechanics. Dexmedetomidine is commonly used for surgical sedation. Whether dexmedetomidine could improve lung mechanics during general anesthesia with desflurane remains unclear. We explored the effects of dexmedetomidine on lung compliance, peak airway pressure, oxygenation index, and postoperative outcomes in patients with desflurane during their surgical procedure in this study.

## 2 Materials and methods

This study was conducted in accordance with the Declaration of Helsinki and approved by the Ethics Committee of Shanghai General Hospital. It has been registered at the Chinese Ethics Committee of Registering Clinical Trials (ChiCTR2100054966). All included patients signed an informed consent form before their surgery. We enrolled patients undergoing lower limb fracture surgery, who received general anesthesia with tracheal intubation in the supine position from January 2022 to October 2022. The inclusion criteria were American Society of Anesthesiologists (ASA) physical status Ⅰ or Ⅱ, age between 18 and 80 years, and body mass index (BMI) ranging from 18 to 28 kg/m^2^. Patients with serious cardiovascular or pulmonary disorders, severe kidney or liver dysfunction, asthma, chronic obstructive pulmonary disease, bronchitis, sinus bradycardia, and shock were excluded.

### 2.1 Randomization

This was a prospective, randomized, double-blind, controlled trial. The participants were randomized using a computer-generated randomization table, with group allocation concealed in sealed opaque envelopes. Patients were assigned to the study by opening the randomization envelopes immediately before the start of anesthesia. The experimental group received Dex (2 mg diluted in 50 mL of 0.9% saline to a final concentration of 4 μg/mL), while the control group received an equal volume of 0.9% saline. The participating anesthetists, nurses, surgeons, and patients were blinded to the treatment allocation. All preoperative and intraoperative management was performed by the same anesthesiologist who was blinded to the study.

### 2.2 Anesthesia management

All patients fasted for 8−12 h before surgery and did not use any preoperative medication. Electrocardiogram, heart rate (HR), non-invasive blood pressure, and pulse oxygen saturation (SpO_2_) were routinely monitored upon arrival at the operation room and patients received sodium lactate ringer solution at a rate of 5 mL/kg/h. A 20-gauge artery catheter (Angiocath, 8608376, H4774-2, BD Medical, Franklin Lake, NJ, USA) was inserted after local anesthesia with lidocaine for real-time blood pressure monitoring and blood gas analysis (Radiometer, Copenhagen, Denmark). After breathing 6 L/min of 100% O_2_ for 5 min, anesthesia was induced by intravenous administration of lidocaine (1.0 mg/kg), sufentanil (0.4 μg/kg), propofol (2 mg/kg), and rocuronium (0.6 mg/kg). When the patients lost consciousness and the muscles were relaxed, an endotracheal tube was intubated under a visual laryngoscope connected to an anesthesia machine (Aelite NTX, GE), and intermittent positive pressure mechanical ventilation was performed. The ventilation mode involved volume control ventilation, a tidal volume of 6–8 mL/kg, a respiratory rate of 10–12 beats/min, an inspiratory-expiratory ratio of 1:1.5, and an end-tidal carbon dioxide partial pressure at 35–45 mmHg. Using a low-flow closed anesthesia system, O_2_ (0.3−0.4 L/min), air (0.2−0.3 L/min), and total fresh gas (0.5−0.6 L/min) were administered, and the fraction of inspired oxygen (FiO_2_) was maintained at approximately 60%. After tracheal intubation, the desflurane was adjusted to a concentration of 18% to obtain an inhaled anesthetic concentration of 1.0 MAC and maintained 6% desflurane during the surgery. A loading dose of 0.25 μg/kg Dex or 0.9% saline was given over 10 min in the two groups before anesthesia induction. Following the conventional induction of tracheal intubation, 0.3 μg/kg/h Dex or 0.9% saline was pumped until the end of the surgery. The depth of anesthesia maintained bispectral index of 40 and 55 in the two groups. Sufentanil (0.1–0.2 μg/kg) and rocuronium (0.1−0.15 mg/kg) were administered at the discretion of the attending anesthetist. All patients were transferred to the post-anesthesia care unit (PACU) after tracheal extubation. Ephedrine was administered if the mean arterial pressure (MAP) decreased by more than 20% from baseline, while atropine was administered if heart rate (HR) was lower than 45 beats per min. The experiment was withdrawn if the peak airway pressure (Ppeak) was higher than 40 cmH_2_O or if the hemodynamics were severely unstable.

Cdyn and Ppeak were continuously monitored using the anesthesia machine (Aelite NTX, GE), an automatic spirometer that evaluated changes through a volume–pressure curve with every breath cycle. Arterial blood gas was analyzed at baseline (T0); the moment after endotracheal intubation was connected to the breathing machine (T1); when 1.0 MAC desflurane was maintained before incision (T2); during incision (T3); and 15 min (T4), 30 min (T5), 60 min (T6), and 90 min (T7) after incision. The partial pressure of O_2_ (PaO_2_) and oxygenation index (OI), expressed as PaO_2_/FiO_2_, were recorded at all time points. Postoperative outcomes including pneumonia and atelectasis, hospitalization days, ICU hospitalization days, and postoperative temperature (within 3 days after surgery) were compared.

### 2.3 Statistical analysis

The primary outcome was dynamic lung compliance (Cdyn) during the surgery. Based on our preliminary findings, the Cdyn was 42.0 ± 0.58 mL/cmH_2_O with intravenous Dex and 38.3 ± 1.16 mL/cmH_2_O with 0.9% normal saline. Forty-four patients (22 patients per group) were required in the study with a two-sided significance level at 0.05 and statistical power of 80% [9]. To account for a 20% dropout rate, we included fifty-six patients (28 patients per group) in our study. The Statistical Package for Social Science (SPSS) software, version 25 for Microsoft Windows (SPSS Inc, Chicago, IL, USA), was used for the data analysis. Continuous data with normal distributions were presented as mean ± standard deviation (SD). Categorical data were presented as frequencies and analyzed using the chi-square test. Variables with repeated measures were analyzed using repeated-measures analysis of variance with post-hoc pairwise comparison using the Bonferroni test. *P* < 0.05 was considered statistically significant.

## 3 Results

Of the 56 patients, five patients were excluded from the study. Two patients refused to participate and three patients (one in the Dex group and two in the control group) were excluded because of incomplete data. Finally, data from 51 patients were analyzed.

The demographic and operation data were detailed in Table 1. There were no significant differences in the baseline characteristics (age, sex ratio, BMI, ASA grade, hypertension, diabetes, smoking history, operation time, fluid infusion volume, and blood loss volume) between the two groups. The hemodynamic parameters were shown in Table 2. In comparison with the control group, MAP and HR revealed no statistical significance in Dex group (*P* = 0.214, 0.443, respectively).

**Table 1:** Demographic and operative data.

	Group		*P-*value
	Dex group (n = 26)	Control group (n = 25)	
Age (years)	59.6 ± 5.45	61.4 ± 7.78	0.360
Sex (male/female)	15/11	11/14	0.328
Weight (kg)	65.6 ± 6.79	64.2 ± 5.67	0.413
Height (cm)	164.8 ± 7.06	162.4 ± 7.39	0.252
BMI (kg/m^2^)	24.2 ± 2.25	24.4 ± 1.88	0.776
ASA grade Ⅰ/Ⅱ (n)	15/11	15/10	0.867
Hypertension (n)	8	7	0.828
Diabetes (n)	3	3	0.959
Smoking (n)	7	4	0.343
Operation time (min)	130.3 ± 10.02	135.0 ± 13.39	0.162
Fluid volume (mL)	1042.3 ± 168.93	996.4 ± 218.31	0.404
Blood loss volume (mL)	83.7 ± 24.96	89.4 ± 29.45	0.452

Values are presented as mean ± standard deviation.

ASA: American Society of Anesthesiologists physical status, BMI: body mass index, Dex: dexmedetomidine.

**Table 2:** Hemodynamic parameters of patients.

	MAP (mmHg)		HR (bpm)	
	Dex	Control	Dex	Control
T0	100.9 ± 6.81	98.8 ± 8.59	74.9 ± 6.63	72.7 ± 7.79
T1	81.2 ± 9.21	83.4 ± 11.32	72.0 ± 6.20	69.4 ± 8.12
T2	75.5 ± 8.29	78.4 ± 9.93	65.8 ± 5.72	67.9 ± 9.56
T3	76.3 ± 7.57	79.6 ± 9.31	66.6 ± 5.99	68.9 ± 5.49
T4	78.3 ± 7.86	81.2 ± 11.43	65.9 ± 5.29	68.2 ± 5.11
T5	78.3 ± 8.38	80.6 ± 7.43	66.5 ± 5.27	68.6 ± 5.87
T6	79.7 ± 6.84	82.4 ± 7.54	66.4 ± 4.72	67.8 ± 6.39
T7	81.6 ± 7.87	84.5 ± 6.20	67.9 ± 4.50	70.3 ± 7.74

Data are shown as mean ± standard deviation.

HR: heart rate, Dex: dexmedetomidine, MAP: mean arterial pressure.

**Fig. 1:**
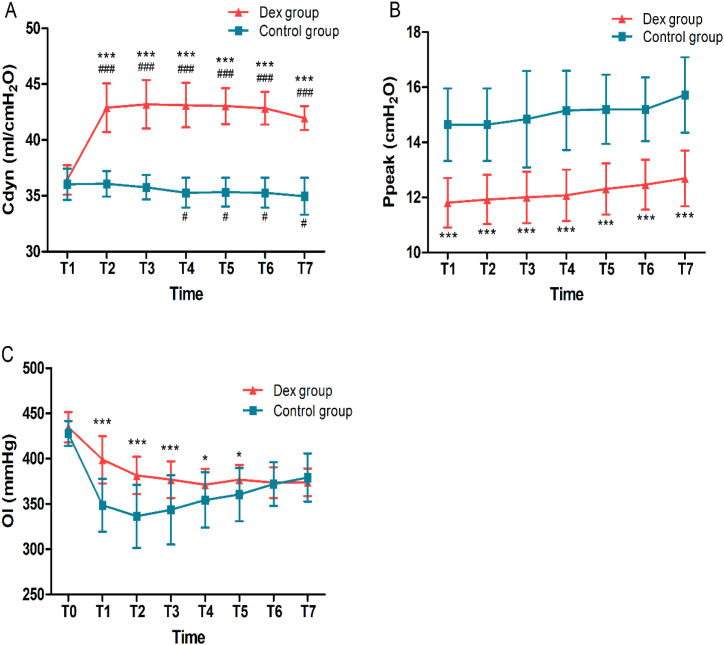
Respiratory parameters of the patients. Changes in the Cdyn (A), Ppeak (B), and OI (C) in the Dex and control groups at different time points. T0, baseline; T1, the moment after endotracheal intubation was connected to the breathing machine; T2, 1.0 minimum alveolar concentration desflurane maintained before incision; T3, during incision; T4, 15 min after incision; T5, 30 min after incision; T6, 60 min after incision; and T7, 90 min after incision. *P < 0.05, ***P < 0.001 compared to the control group, ^#^*P* < 0.05, ^###^*P* < 0.001 compared to T1. Cdyn: dynamic lung compliance, Dex: dexmedetomidine, OI: oxygenation index, PaO_2_/FiO_2_: ratio of arterial oxygen partial pressure to fractional inspired oxygen, Ppeak: peak airway pressure.

Fig. 1 detailed the comparison between the respiratory parameters of the two groups. There was no significant between-group difference in the Cdyn at T1 between the two groups (36.4 ± 1.33 vs. 36.0 ± 1.34 mL/cmH_2_O, *P* = 0.321). Compared with the control group, the Dex group showed a higher Cdyn at T2, T3, T4, T5, T6, and T7 (all *P* < 0.001). In the control group, Cdyn at T4, T5, T6, and T7 were lower than that at T1 (*P* = 0.032, 0.043, 0.032 and 0.018, respectively) (Fig. 1A). Ppeak was significantly lower in the Dex group than that at all time points in the control group (all *P* < 0.001) (Fig. 1B).

Results of OI were shown in Fig. 1. While breathing room air, there was no significant difference between the two groups in the baseline OI at T0 (434.8 ± 16.76 vs. 427.9 ± 13.94 mmHg, *P* = 0.115). Compared to the control group, the OI expressed as PaO_2_/FiO_2_ ratio was significantly higher in the Dex group at T1 (399.0 ± 26.20 vs. 348.5 ± 29.24 mmHg, *P* < 0.001), T2 (381.5 ± 20.64 vs. 336.5 ± 34.91 mmHg, *P* < 0.001), T3 (376.9 ± 20.28 vs. 343.6 ± 38.29 mmHg, *P* < 0.001), T4 (371.3 ± 17.59 vs. 354.5 ± 30.67 mmHg, *P* = 0.02) and T5 (376.9 ± 16.26 vs. 360.4 ± 29.43 mmHg, *P* = 0.016) (Fig. 1C).

Table 3 listed the postoperative outcomes. In the PACU (3L/min O_2_ with a nasal catheter), the SpO_2_ was ≥98% in all patients in both groups. In comparison, no patient from the two groups required admission to the ICU for respiratory support therapy after surgery. The two groups showed no significant differences in hospitalization days (3.5 ± 0.81 vs. 3.8 ± 0.76 days, *P* = 0.125) or body temperature (3-day after surgery) (*P* = 0.068, 0.233, 0.489, respectively). According to the outpatient records, no patient was diagnosed with a postoperative lung infection, and there were no significant differences in postoperative outcomes between the two groups.

**Table 3:** Postoperative outcomes.

	Group		*P-*value
	Dex group (n = 26)	Control group (n = 25)	
SpO_2_ at PACU (%)	98.7 ± 1.00	98.8 ± 1.00	0.710
Intensive care unit stay (days)	0	0	1.000
Atelectasis (n)	0	0	1.000
Postoperative lung infection (n)	0	0	1.000
Hospitalization day (days)	3.5 ± 0.81	3.8 ± 0.76	0.125
Temperature Day 1 (°C)	36.7 ± 0.32	36.6 ± 0.21	0.068
Day 2 (°C)	36.6 ± 0.21	36.5 ± 0.23	0.233
Day 3 (°C)	36.6 ± 0.23	36.6 ± 0.25	0.489

Data are shown as mean ± standard deviation.

Dex: dexmedetomidine, PACU: post-anesthesia care unit, SpO_2_: pulse oxygen saturation.

## 4 Discussion

This is the first prospective randomized study to evaluate the effects of dexmedetomidine on lung mechanics, oxygenation index and postoperative outcomes under general anesthesia with desflurane. Improving lung compliance and avoiding high airway resistance during general anesthesia with desflurane was essential. In this study, we found that Dex group showed higher Cdyn, lower peak airway pressure and higher oxygenation index compared with the control group.

In phase III clinical trials, the most common adverse events associated with Dex are hypotension, tachycardia, and nausea [7]. In our study, hypotension and reduced heart rate were evident in the Dex group; however, no significant differences were found between the two groups. Potts et al. [10] recommended a small dose of Dex (0.5 μg/kg) for hemodynamic stability. In this trial, a low dose of 0.3 μg/kg/h Dex was administered consistently through a pump injection until the end of surgery to avoid cardiovascular side effects. Although this dose of Dex could reduce the MAP and HR, it was still within the clinically acceptable range and did not cause serious hemodynamic effects in the patients. In addition, 100% oxygen absorbed by the small closed respiratory tract causes atelectasis and pulmonary shunt. Compared to 60% oxygen concentration, a concentration of 100% oxygen may result in larger atelectasis of lung tissue [11]. Meanwhile, none of the patients in this study had a SpO_2_ < 95%.

Although desflurane has certain clinical advantages, its use is limited to patients with specific airway hyperresponsiveness. Sivaci et al. [12] found that lung compliance was significantly reduced in patients following mechanical ventilation with desflurane inhalation for 5 min before undergoing laparoscopic gynecological surgery. Zhou et al. [13] have further demonstrated that the bronchoconstriction effect of desflurane was related to the significantly increased concentrations of cyclic adenosine monophosphate in the airway smooth muscles. However, no significant bronchial stenosis was shown at 0.5 and 1.0 MAC desflurane. Additional, in our study, we found lower Cdyn during general anesthesia with desflurane in control group. Studies in sheep on general anesthesia have shown an immediate decrease in Cdyn following intravenous administration of Dex. A significant decrease may have been associated with direct activation of postsynaptic α_2_-adrenal receptors of the bronchus or indirect bronchospasm caused by alkaline activation [14]. However, other studies have shown that Dex has a protective effect against lipopolysaccharide-induced lung injury in rats [15]. Moreover, Dex administered through the vein alleviates histamine-induced reflex bronchoconstriction in canine airways [16]. It also inhibits interleukin-6 release and reduces mechanical ventilator-related lung injury and endothelium-induced inflammatory responses [17]. Several clinical studies in patients with single-lung ventilation have shown that Dex does not increase pulmonary artery pressure in patients with pre-existing pulmonary hypertension [18]. Furthermore, dexmedetomidine has been shown to improve lung compliance and oxygenation while reducing dead space in patients with chronic obstructive pulmonary disease during lung cancer surgery [16]. Lung compliance refers to the change in lung volume in response to the change in unit pressure and is a sensitive indicator of lung ventilation and lung injury. High lung compliance may reflect a lower airway response to a certain extent. It is necessary to improve lung compliance in patients under general anesthesia, reduce respiratory work, and promote gas exchange. In our study, we excluded patients with pulmonary and airway diseases, baseline pulmonary function was not as routine pre-operative examination. Our study found that 0.25 μg/kg followed by maintenance with 0.3 μg/kg/h dexmedetomidine significantly improved lung compliance in Dex group. Dex-mediated inhibition of the adverse effects of tracheal intubation and stabilization of the respiratory state during surgery might account for the high Cdyn following Dex administration.

A study on awake patients with moderate asthma who underwent bronchoscopy found that endotracheal intubation decreased the 1-s forced expiratory volume by 50%. In comparison, α_2_ agonists attenuated the tracheal intubation response by 10%, suggesting that Dex can be used for severe asthma or high airway reactions [19]. Dex may attenuate airway hyperresponsiveness by inhibiting the TLR4/NF-κB pathway in mice [20]. The Ppeak is proportional to airway resistance. Our study also demonstrated that compared to the control group, the Dex group had a significantly lower Ppeak.

The OI reflects the lung function of gas exchange; the larger the OI, the better the pulmonary ventilation function. The physiological effects of single-lung ventilation often lead to oxygenation dysfunction. A meta-analysis of patients who underwent thoracic surgery with one-lung ventilation indicated that Dex, compared to saline controls, significantly promoted an OI increase in these patients; moreover, it reduced pulmonary shunt by increasing blood nitric oxide levels and increased PaO_2_ [21]. At the same time, inhaled anesthetics can inhibit hypoxic pulmonary vasoconstriction, increase perfusion of the ventilated lung and nitric oxide levels, and reduce intrapulmonary shunt [7]. Dex infusion can improve lung compliance and OI (increased by 10% after 90 min) with restrictive ventilation dysfunction in patients undergoing bariatric surgery [22]. In our study, compared to the control group, the Dex group maintained a higher OI during the intraoperative procedure. Clinically, it has been proven that Dex can cause a certain degree of sedation in patients undergoing surgery [23]. We also found no significant differences between the two groups in the postoperative outcomes, including length of hospital stay, postoperative body temperature and postoperative lung complications.

Despite the novel findings of the present trial, our study had some limitations. First, inflammatory factors and mechanisms underlying the effects of Dex on pulmonary vessels were not considered. Second, the sample size of the study was small. Clinical trials with larger sample sizes are warranted to confirm our results. Third, a low dose of Dex was administered in this study. The effects of larger doses need further exploration. Fourth, although the study evaluated the effects of Dex at several time points, its effects during prolonged surgeries need to be examined. Finally, preoperative pulmonary function of patients was not evaluated in this study.

In conclusion, our study showed that a loading dose of 0.25 μg/kg followed by maintenance with 0.3 μg/kg/h Dex infusion improved dynamic lung compliance in patients maintained with desflurane during general anesthesia.

## Author contribution statement

Xiaoli Wang, Chao Gong: Performed the experiments; Analyzed and interpreted the data; Contributed reagents, materials, analysis tools or data; Wrote the paper.

Yi Zhang: Performed the experiments; Analyzed and interpreted the data; Contributed reagents, materials, analysis tools or data.

Shitong Li, Lina Huang, Lianhua Chen: Conceived and designed the experiments; Contributed reagents, materials, analysis tools or data.

## Data availability statement

Data included in article/supp. material/referenced in article.

## Funding statement

This research did not receive any specific grant from funding agencies in the public, commercial, or not-for-profit sectors.

## Declaration of competing interest

The authors declare that they have no known competing financial interests or personal relationships that could have appeared to influence the work reported in this paper.
